# The safety attitudes questionnaire – ambulatory version: psychometric properties of the Norwegian translated version for the primary care setting

**DOI:** 10.1186/1472-6963-14-139

**Published:** 2014-03-29

**Authors:** Gunnar Tschudi Bondevik, Dag Hofoss, Elisabeth Holm Hansen, Ellen Catharina Tveter Deilkås

**Affiliations:** 1 Research Group for General Practice, Department of Global Public Health and Primary Care, University of Bergen, Bergen, Norway; 2 National Centre for Emergency Primary Health Care, Uni Health, Uni Research, Bergen, Norway; 3 Institute of Health and Society, University of Oslo, Oslo, Norway; 4 Haraldsplass Deaconess University College, Bergen, Norway; 5 Norwegian Knowledge Centre for the Health Services, Olso, Norway; 6 Health Services Research Unit, Akershus University Hospital, Lørenskog, Norway

**Keywords:** Adverse events, General practice, Medical errors, Out-of-hours, Patient safety culture, Primary care, Quality improvement, Safety attitudes questionnaire

## Abstract

**Background:**

Patient safety culture is how leader and staff interaction, attitudes, routines and practices protect patients from adverse events in healthcare. The Safety Attitudes Questionnaire is the most widely used instrument to measure safety attitudes among health care providers. The instrument may identify possible weaknesses in clinical settings, and motivate and guide quality improvement interventions and reductions in medical errors. The Safety Attitudes Questionnaire – Ambulatory Version was developed for measuring safety culture in the primary care setting. The original version includes six major patient safety factors: Teamwork climate, Safety climate, Job satisfaction, Perceptions of management, Working conditions and Stress recognition. We describe the results of a validation study using the Norwegian translation of the questionnaire in the primary care setting, and present the psychometric properties of this version.

**Methods:**

The study was done in seven Out-of-hours casualty clinics and 17 regular GP practices employing a total of 510 primary health care providers (194 nurses and 316 medical doctors). In October and November 2012, the translated Safety Attitudes Questionnaire – Ambulatory Version was distributed by e-mail. Data were collected electronically using the program QuestBack, whereby the participants responded anonymously. SPSS was used to estimate the Cronbach’s alphas, item-to-own-factor correlations, intercorrelations of factors and item-descriptive statistics. The confirmatory factor analysis was done by AMOS.

**Results:**

Of the 510 invited health care providers, 266 (52%) answered the questionnaire - 72% of the registered nurses (n = 139) and 39% of the medical doctors (n = 124). In the confirmatory factor analysis, the following five factor model was shown to have acceptable goodness-of-fit values in the Norwegian primary care setting: Teamwork climate, Safety climate, Job satisfaction, Working conditions and Perceptions of management.

**Conclusions:**

The results of our study indicate that the Norwegian translated version of the Safety Attitudes Questionnaire – Ambulatory Version, with the five confirmed factors, might be a useful tool for measuring several aspects of patient safety culture in the primary care setting. Further research should investigate whether there is an association between patient safety culture in primary care, as measured by the Safety Attitudes Questionnaire – Ambulatory Version, and occurrence of medical errors and negative patient outcome.

## Background

Over the last years, there has been an increasing focus on medical errors and patient safety. Until recently, patient safety issues have mainly been addressed in the hospital care setting [1-7]. However, adverse events are common also in primary care, where the largest volume of health care is delivered. For these reasons, there has been an increasing interest in factors related to patient safety also outside the hospital setting.

Patient safety culture is how leader and staff interaction, attitudes, routines and practices protect patients from adverse events in healthcare [8]. The concept is developed within the framework and research of organizational psychology, and is regarded as a group phenomenon rather than that of an individual.

A number of instruments have been developed to measure safety attitudes of health care providers [1,9-13]. The Safety Attitudes Questionnaire (SAQ) is most widely used, and the original version includes six major patient safety factors: Teamwork climate, Safety climate, Job satisfaction, Perceptions of management, Working conditions and Stress recognition [14].

SAQ scores have been shown to correlate with patient outcome in care giving units [12,15-17]. The instrument may identify possible weaknesses in a clinical setting and motivate quality improvement interventions and reductions in medical errors [18-20]. The SAQ has been translated and validated in a number of different countries, including Norway [17].

The majority of the safety attitudes instruments are adapted to different clinical settings within hospitals. It has been shown that patient safety culture may vary substantially across hospitals, departments and - in particular - wards, the level closest to the patients [21]. Interventions to improve patient safety should therefore also include ward level.

In 2007, the first questionnaire for measuring safety culture in the outpatient setting was described [1]. This Safety Attitudes Questionnaire – Ambulatory Version (SAQ-AV) was developed from the original SAQ, and adjusted to the primary care setting. It has shown to be a reliable tool for comparing attitudes across different professional groups of health care providers outside hospitals [1]. Further, SAQ-AV gives data that makes it possible to focus on patient safety improvement activities, in addition to measuring change in attitudes over time [22].

In this paper, we describe the results of a validation study using the Norwegian translation of the SAQ-AV in the primary care setting, both in out-of-hours (OOH) casualty clinics and regular General Practitioner (GP) practices. We wanted to study whether the factor structure in the translated version was the same as in the original questionnaire. We present the psychometric properties of the Norwegian translated version of the SAQ-AV. The study is a part of the Norwegian patient safety campaign “In Safe Hands”, which was launched in 2011 by the Ministry of Health.

## Methods

### Material

The present study was done both in OOH casualty clinics and in regular GP practices. Seven OOH clinics in Norway function as especially designated “Watchtower Clinics”, established by the National Centre for Emergency Primary Health Care to deliver research data [23,24]. The seven Watchtowers were selected so as to be representative for OOH casualty clinics in Norway, and serve 4,6% (226,000 inhabitants) of the nations population in a total of 18 municipalities. In addition, all regular GP practices in Sogn & Fjordane County were invited to participate in the study. This is one of 19 counties in Norway, with a population of approximately 110,000 in 26 municipalities.

In order to protect the confidentiality of the respondents, we only included clinics and practices employing at least five health professionals having clinical patient contact. For this reason we replaced one of the seven Watchtowers with the OOH clinic in the neighbouring municipality. The seven OOH clinics participating in our study employed a total of 337 health professionals, of whom 231 medical doctors and 106 nurses. They serve a total population of 251,000.

Seven of the total of 30 regular GP practices in Sogn & Fjordane County were not included, as they had less than five employees working clinically. Of the remaining 23 regular GP practices, 17 agreed to participate in the study. These 17 regular GP practices employed a total of 173 health professionals, 85 medical doctors and 88 support medical staff. The professional background of the support medical staff varied, and included registered nurses, medical secretaries and bioengineers. In this paper, we use the term “nurses” for this group of support medical staff. The participating GP clinics serve a population of 70,000.

### Translation procedures

The original SAQ-AV questionnaire was translated following modified principles adapted from Beaton et al. [25]. Initially, the original English version was translated into Norwegian using a professional translation bureau. Next, an expert committee with clinicians and researchers from the Research Group for General Practice (University of Bergen), the National Centre for Emergency Primary Health Care (Uni Research, Bergen) and the Norwegian Knowledge Centre for the Health Services (Oslo) adapted the initial translated version to the primary care setting in Norway. This adapted version of the questionnaire was translated back into English by a second independent translation bureau being blinded to the original version.

Based on this back-translated version, the expert committee made some adjustments in order to clarify misunderstandings. The prefinal Norwegian version was tested in a group of primary health care providers. Based on their feedback, the final version of the translated questionnaire was developed. Two Norwegian versions of the questionnaire were made, one for OOH casualty clinics and one for regular GP practices, with only minor modifications in a few of the questions – according to the setting. Pre-tests showed that it took approximately 15 min to complete the SAQ-AV questionnaire.

### Scoring

The SAQ-AV is a 62 item questionnaire where the respondents rate their agreement using a 5-point Likert scale: 1 = disagree strongly, 2 = disagree slightly, 3 = neutral, 4 = agree slightly, 5 = agree strongly. For all questions, “Not applicable” was included as a response category, and combined with missing values in the data analyses. Scores of negatively worded items were reversed, so that higher scores in the data set always indicate a more positive evaluation of the unit’s patient safety culture.

### Data collection

The seven OOH clinics and 17 regular GP practices provided the e-mail addresses of all employees having direct patient contact in their clinical work. In October and November 2012, the SAQ-AV was distributed by e-mail to all 510 primary health care providers in these 24 clinics/practices (316 medical doctors and 194 nurses). Data were collected electronically using the program QuestBack, whereby the participants responded anonymously. This program automatically sent a reminder to those who had not responded after two weeks. After four weeks, an additional reminder was sent to the administrative key persons in the OOH clinics and regular GP practices – asking them to motivate the clinical staff to participate in the study.

After the study, each of the participating OOH clinics and GP practices received a summary of the SAQ-AV results for their own unit. In this way, the health care providers were encouraged to focus on specific factors related to patient safety, and to discuss possible strategies for improvement within their clinical setting.

### Statistical analysis

The QuestBack file with anonymous SAQ-AV data was converted into a SPSS file for further analysis. SPSS v.18 was used to estimate the Cronbach’s alphas, item-to-own-factor correlations, intercorrelations of factors and item-descriptive statistics. The confirmatory factor analysis was done by AMOS.

### Internal consistency

Cronbach’s alpha is a measure of factor score consistency. The test was done to demonstrate to which extent the responses of items within a factor correlated pairwise. Cronbach’s alpha scores above 0.7 were considered adequate. Item-to-own-factor correlations were checked to see if the items correlated more with the factor they were hypothesised to belong to, than to the other factors.

### Hypothesised factor structure

The original SAQ, developed at the University of Texas at Austin [14], described six factors: Teamwork climate, Safety climate, Working conditions, Job satisfaction, Perceptions of management and Stress recognition (Table 1). Only 30 of the 62 items in the SAQ-AV are covered by the original six factors. The rest of the items were considered useful for local improvement processes and discussion and included to provide additional information regarding the work environment in the primary care setting. Since some of these items belonged thematically to the Teamwork climate factor (Q37, Q39, Q45) we included the items in the hypothesized factor structure.

**Table 1:** The six factors and corresponding items in the original Safety Attitudes Questionnaire (SAQ) version

**Teamwork climate**	Nurse input is well received in this office.
	In this office, it is difficult to speak up if I perceive a problem with patient care.
	Disagreements in this office are resolved appropriately (i.e., not *who* is right but *what* is best for the patient).
	I have the support I need from other personnel to care for patients.
	It is easy for personnel in this office to ask questions when there is something that they do not understand.
	The physicians and nurses here work together as a well-coordinated team.
**Safety climate**	I would feel safe being treated here as a patient.
	Medical errors are handled appropriately in this office.
	I receive appropriate feedback about my performance.
	In this office, it is difficult to discuss errors.
	I am encouraged by my colleagues to report any patient safety concerns I may have.
	The culture in this office makes it easy to learn from the errors of others.
	I know the proper channels to direct questions regarding patient safety in this office.
**Working conditions**	This office does a good job of training new personnel.
	All the necessary information for diagnostic and therapeutic decisions is routinely available to me.
	This office deals constructively with problem personnel.
	Trainees in my discipline are adequately supervised.
**Job satisfaction**	I like my job.
	Working in this office is like being part of a large family.
	This office is a good place to work.
	I am proud to work at this office.
	Morale in this office is high.
**Perceptions of management**	The management of this office supports my daily efforts.
	Office management does not knowingly compromise the safety of patients.
	The levels of staffing in this office are sufficient to handle the number of patients.
	I am provided with adequate, timely information about events in the office that might affect my work.
**Stress recognition**	When my workload becomes excessive, my performance is impaired.
	I am less effective at work when fatigued.
	I am more likely to make errors in tense or hostile situations.
	Fatigue impairs my performance during emergency situations (e.g. code or cardiac arrest).

One item (Q18) had been moved from the factor Perceptions of management to the factor Working conditions, in the validation of the Norwegian SAQ Short form 2006 [17]. We replicated this since we also in the present study found that the item (Q18) fitted the Working condition factor better, indicating that respondents in primary care perceive that adequate staffing has less to do with leadership, although it is strongly related to working conditions. Since several studies find that the factor Stress recognition does not vary significantly between organizational units [21,26], and also has problems regarding construct validity [27], it cannot be considered a valid factor for measuring patient safety [28]. In the present study, we based our analyses on the five remaining factors, which were included in the hypothesised factor model.

### Confirmatory factor analysis (CFA)

Among the 160 respondents answering each of the items (no missing/not applicable), we tested the hypothesized factors from the five factor model of the SAQ using AMOS. The factors reflect the correlation structure in the item responses. Valid factors should thus reflect a thematic logic that is coherent with the purpose of the questionnaire. CFA provides goodness-of-fit indices, which show how the survey responses comply with the pre-hypothesised factor model.

The following goodness-of-fit indices were calculated: P, Pclose, Adjusted Goodness-of-Fit Index (AGFI), Comparative Fit Index (CFI), Normed Fit Index (NFI), Root Mean Square Error of Approximation (RMSEA) and Hoelter 0.05. Acceptable goodness-of-fit values indicate that the SAQ-AV measures patient safety culture in the hypothesised factors. The P and Pclose values should exceed 0.05, the AGFI should be close to 1, and the RMSEA should not exceed 0.10 [29]. The CFI should be close to 1 and the NFI > 0.90 [30]. The Hoelter 0.05 should exceed 200, an estimate of the largest sample for which a data set with these intercorrelations among the variables would confirm the model [31].

### Ethical considerations

This study was based on data regarding patient safety culture among health care providers. It was conducted in compliance with the ethical guidelines of the Helsinki Declaration. All participants received written information about the purpose of the study, and that the data would be collected anonymously and treated in confidence. As the study was not affected by the Norwegian Health Research Act, approval from an ethics committee was not needed. The study was approved by the Norwegian Social Science Data Services – the governmental agency for protecting survey research respondent privacy according to the Norwegian Personal Data Act (Ref. No. 2012/30774).

## Results

Of the 510 invited health care providers, 266 (52%) answered the questionnaire - 72% of the nurses (n = 139) and 39% of the medical doctors (n = 124). Professional status is not known for three of the respondents. The response rate was higher among doctors in GP practices (55%) than doctors in OOH clinics (33%), while the corresponding rates for nurses were 73% and 71%, respectively.

Table 2 presents mean scores with standard deviations for each of the 62 items, expressing the degree of agreement with the statements in the questionnaire (1 = disagree strongly, 2 = disagree slightly, 3 = neutral, 4 = agree slightly, 5 = agree strongly). Missing values at item levels are also shown in the table, and were on average 2.4%, ranging from 0 to 8.3%. The strongest disagreement was found in the statements “I feel burned out from my work” and “Abnormal test results are frequently lost or overlooked” (both mean scores 1.7). The highest mean scores reflecting agreement were reported for the statements “I like my job” and “Attending physicians/primary care providers in this office are doing a good job” (both mean scores 4.7).

**Table 2:** Mean score for the 62 items in the Safety Attitudes Questionnaire – Ambulatory Version (SAQ-AV)

**Statement**	**Missing/NA**^**a**^**n (%)**	**Mean score**^**b**^**(SD**^**c**^**)**
1. High levels of workload are common in this office.	0 (0)	4.4 (0.9)
2. I like my job.	1 (0.4)	4.7 (0.7)
3. Nurse input is well received in this office.	6 (2.3)	4.3 (1.0)
4. I would feel safe being treated here as a patient.	3 (1.1)	4.5 (0.8)
5. Medical errors are handled appropriately in this office.	5 (1.9)	4.1 (0.9)
6. This office does a good job of training new personnel.	5 (1.9)	3.9 (1.1)
7. All the necessary information for diagnostic and therapeutic decisions is routinely available to me.	14 (5.3)	4.2 (0.9)
8. Working in this office is like being part of a large family.	3 (1.1)	3.8 (1.1)
9. Senior management of this office is doing a good job.	11 (4.1)	4.2 (1.0)
10. The management of this office supports my daily efforts.	15 (5.6)	4.1 (1.0)
11. I receive appropriate feedback about my performance.	2 (0.8)	3.5 (1.2)
12. In this office, it is difficult to discuss errors.	2 (0.8)	2.1 (1.1)
13. Briefing other personnel before a procedure (e.g., biopsy) is important for patient safety.	11 (4.1)	4.1 (1.1)
14. Briefings are common in this office.	7 (2.6)	3.2 (1.2)
15. This office is a good place to work.	2 (0.8)	4.6 (0.7)
16. Communication breakdowns which lead to delays in delivery of care are common.	1 (0.4)	2.1 (1.1)
17. Office management does not knowingly compromise the safety of patients.	13 (4.9)	4.2 (1.3)
18. The levels of staffing in this office are sufficient to handle the number of patients.	1 (0.4)	3.5 (1.4)
19. Decision making in this office utilizes input from relevant personnel.	9 (3.4)	4.1 (1.0)
20. I am encouraged by my colleagues to report any patient safety concerns I may have.	7 (2.6)	3.8 (1.2)
21. The culture in this office makes it easy to learn from the errors of others.	4 (1.5)	3.5 (1.2)
22. This office deals constructively with problem personnel.	7 (2.6)	3.6 (1.0)
23. The medical equipment in this office is adequate.	5 (1.9)	4.0 (1.1)
24. In this office, it is difficult to speak up if I perceive a problem with patient care.	3 (1.1)	2.0 (1.1)
25. When my workload becomes excessive, my performance is impaired.	4 (1.5)	3.6 (1.2)
26. I am provided with adequate, timely information about events in the office that might affect my work.	4 (1.5)	3.8 (1.2)
27. I have seen others make errors that had the potential to harm patients.	8 (3.0)	2.4 (1.3)
28. I know the proper channels to direct questions regarding patient safety in this office.	2 (0.8)	4.1 (1.2)
29. I am proud to work at this office.	1 (0.4)	4.4 (0.8)
30. Disagreements in this office are resolved appropriately (i.e., not *who* is right but *what* is best for the patient).	2 (0.8)	4.0 (0.9)
31. I am less effective at work when fatigued.	0 (0)	4.4 (0.8)
32. I am more likely to make errors in tense or hostile situations.	9 (3.4)	4.2 (0.9)
33. Stress from personal problems adversely affects my performance.	9 (3.4)	3.5 (1.2)
34. I have the support I need from other personnel to care for patients.	5 (1.9)	4.5 (0.8)
35. It is easy for personnel in this office to ask questions when there is something that they do not understand.	0 (0)	4.6 (0.8)
36. Disruptions in the continuity of care can be detrimental to patient safety.	13 (4.9)	4.0 (1.1)
37. During emergencies, I can predict what other personnel are going to do next.	11 (4.1)	3.7 (0.9)
38. The physicians and nurses here work together as a well-coordinated team.	3 (1.1)	4.1 (1.0)
39. I am frequently unable to express disagreement with staff physicians/intensivists in this office.	11 (4.1)	2.5 (1.2)
40. Truly professional personnel can leave personal problems behind when working.	6 (2.3)	3.9 (1.1)
41. Morale in this office is high.	0 (0)	4.5 (0.7)
42. Trainees in my discipline are adequately supervised.	8 (3.0)	4.0 (1.0)
43. I know the first and last names of all the personnel I worked with during my last shift.	2 (0.8)	4.0 (1.5)
44. I have made errors that had the potential to harm patients.	5 (1.9)	2.6 (1.4)
45. Attending physicians/primary care providers in this office are doing a good job.	1 (0.4)	4.7 (0.6)
46. All the personnel in this office take responsibility for patient safety.	2 (0.8)	4.4 (0.8)
47. I feel fatigued when I have to get up in the morning and face another day on the job.	9 (3.4)	2.1 (1.3)
48. Patient safety is constantly reinforced as the priority in this office.	5 (1.9)	4.1 (1.0)
49. I feel burned out from my work.	3 (1.1)	1.7 (1.1)
50. Important issues are well communicated at shift changes.	7 (2.6)	4.0 (1.1)
51. There is widespread adherence to clinical guidelines and evidence-based criteria in this office.	11 (4.1)	3.8 (0.9)
52. I feel frustrated by my job.	1 (0.4)	1.8 (1.1)
53. I feel I am working too hard on my job.	1 (0.4)	2.3 (1.3)
54. Information obtained through incident reports is used to make patient care safer in this office.	8 (3.0)	3.7 (1.3)
55. Personnel frequently disregard rules or guidelines (e.g., hand washing, treatment protocols/clinical pathways, sterile fields, etc.) that are established for this office.	6 (2.3)	2.0 (1.1)
56. Fatigue impairs my performance during emergency situations (e.g. code or cardiac arrest).	16 (6.2)	2.4 (1.4)
57. Fatigue impairs my performance during routine care.	9 (3.4)	2.8 (1.4)
58. I am satisfied with the current referral process in this office.	15 (5.6)	4.0 (1.0)
59. There is adequate and timely transfer of patient information between the primary care physician and the specialist.	12 (4.5)	4.0 (1.0)
60. Medications are refilled in a timely manner.	12 (4.5)	4.3 (0.9)
61. Medications are refilled correctly.	22 (8.3)	4.5 (0.8)
62. Abnormal test results are frequently lost or overlooked.	8 (3.0)	1.7 (1.0)

^a^NA = Not applicable.

^b^Scoring: 1 = disagree strongly, 2 = disagree slightly, 3 = neutral, 4 = agree slightly, 5 = agree strongly.

^c^Standard deviation.

Results based on answers from 266 primary health care providers working in 7 OOH casualty clinics and 17 GP practices.

The confirmatory factor analysis showed that each of the five hypothesised factors (Teamwork climate, Safety climate, Job satisfaction, Working conditions and Perceptions of management) fitted the data well. The goodness-of-fit indices for the five factors are presented in Table 3. As the P was satisfactory for all factors (p > 0.05), Pclose is not presented in the table.

**Table 3:** Goodness of fit indices for patient safety culture factors among 160* primary health care providers

	**P**	**AGFI**	**CFI**	**NFI**	**RMSEA**	**Hoelter 05**
**Teamwork climate**	0.41	0.94	1.00	0.93	0.02	228
**Safety climate**	0.66	0.96	1.00	0.95	< .001	334
**Job satisfaction**	0.17	0.95	0.99	0.98	0.06	259
**Working conditions**	0.12	0.94	0.98	0.96	0.06	235
**Perceptions of management**	0.18	0.95	0.99	0.96	0.05	268

P: should exceed 0.05.

AGFI: Adjusted Goodness-of-Fit Index, should be close to 1.

CFI: Comparative Fit Index, should be close to 1.

NFI: Normed Fit Index, should exceed 0.90.

RMSEA: Root Mean Square Error of Approximation, should not exceed 0.10.

Hoelter 0.05: should exceed 200.

*160 of the 266 primary health care providers working in the 7 OOH casualty clinics and 17 GP practices included in this study replied to each of the items (they had no missing/not applicable in any of the items related to the five factors).

**Figure 1:**
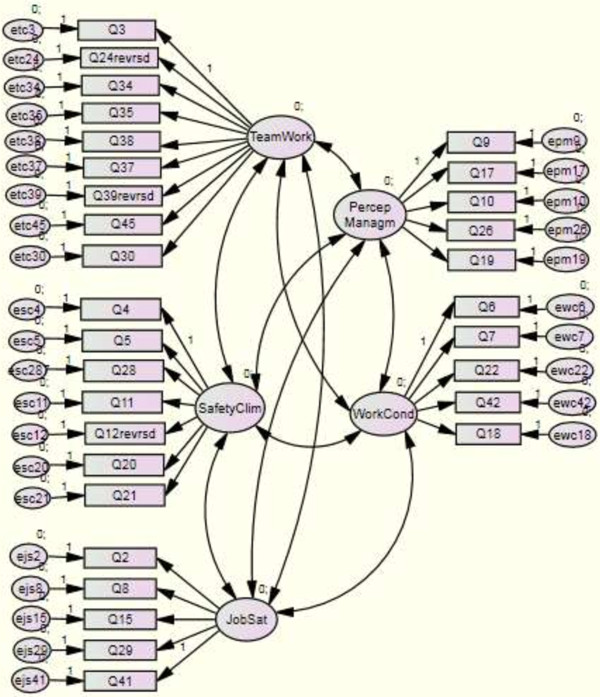
Factor structure model.

The goodness of fit of the entire five-factor model is presented in Figure 1. It is not as clearcut as that of the five single factor models: The CFI of the entire model was 0.86, its NFI was 0.73 and its P-value (and its Pclose-value) was < 0.001. However, its RMSEA was satisfactory (0.07), and the model’s chisquare-to-degrees-of-freedom ratio was excellent: 1.82.

The Cronbach alphas ranged from 0.67 to 0.83 for the factor scores Teamwork climate, Safety climate, Working conditions, Job satisfaction and Perceptions of management (Table 4). All items in the table correlated higher with the factor it has been related to than with any other factor.

**Table 4:** **Item variation and internal consistency of five factors based on 160**^**a**^**respondents**

**Teamwork climate: Cronbach’s alpha: 0.83**	**Mean (SD**^**b**^**)**	**ITOFC**^**c**^
3. Nurse input is well received in this office.	4.23 (1.01)	0.62
24^**d**^. In this office it is difficult to speak up if I perceive a problem with patient care.	4.00 (1.15)	0.53
30. Disagreements in this office are resolved appropriately (i.e., not who is right but what is best for the patient).	3.97 (0.97)	0.62
34. I have the support I need from other personnel to care for patients.	4.45 (0.82)	0.65
35. It is easy for personnel in this office to ask questions when there is something that they do not understand.	4.56 (0.77)	0.61
37. During emergencies, I can predict what other personnel are going to do next.	3.69 (0.88)	0.36
38. The physicians and nurses here work together as a well-coordinated team.	4.02 (0.97)	0.62
39^**d**^. I am frequently unable to express disagreement with staff physician/ intensivists in this office.	3.50 (1.44)	0.41
45. Attending physicians/primary care providers in this office are doing a good job.	4.73 (0.49)	0.52
**Safety climate: Cronbach’s alpha: 0.77**	**Mean (SD**^**b**^**)**	**ITOFC**^**c**^
4. I would feel safe being treated here as a patient.	4.54 (0.74)	0.27
5. Medical errors are handled appropriately in this office.	4.12 (0.88)	0.60
11. I receive appropriate feedback about my performance.	3.39 (1.16)	0.56
12^**d**^. In this office, it is difficult to discuss errors.	3.84 (1.09)	0.58
20. I am encouraged by my colleagues to report any patient safety concerns I may have.	3.80 (1.21)	0.55
21. The culture in this office makes it easy to learn from the errors of others.	3.41 (1.18)	0.63
28. I know the proper channels to direct questions regarding patient safety in this office.	4.06 (1.19)	0.46
**Job satisfaction: Cronbach’s alpha: 0.74**	**Mean (SD**^**b**^**)**	**ITOFC**^**c**^
2. I like my job.	4.75 (0.61)	0.50
8. Working in this office is like being part of a large family.	3.75 (1.13)	0.49
15. This office is a good place to work.	4.61 (0.72)	0.72
29. I am proud to work at this office.	4.46 (0.80)	0.62
41. Morale in this office is high.	4.42 (0.76)	0.32
**Working conditions: Cronbach’s alpha: 0.71**	**Mean (SD**^**b**^**)**	**ITOFC**^**c**^
6. This office does a good job at training new personnel.	3.85 (1.09)	0.64
7. All the necessary information for diagnostic and therapeutic decisions is routinely available to me.	4.18 (0.94)	0.55
18. The levels of staffing in this office are sufficient to handle the number of patients.	3.41 (1.45)	0.56
22. This office deals constructively with problem personnel.	3.50 (0.99)	0.55
42. Trainees in my discipline are adequately supervised.	3.91 (1.05)	0.63
**Perceptions of management: Cronbach’s alpha: 0.67**	**Mean (SD**^**b**^**)**	**ITOFC**^**c**^
9. Senior management of this office is doing a good job.	4.16 (1.03)	0.59
10. The management of this office supports my daily efforts.	4.09 (1.00)	0.59
17. Office management does not knowingly compromise the safety of patients.	4.20 (1.29)	0.25
19. Decision making in this office utilizes input from relevant personnel.	4.06 (0.99)	0.36
26. I am provided with adequate, timely information about events in the office that might affect my work.	3.72 (1.12)	0.39

^**a**^160 of the 266 health care providers included in this study replied to each of the items (they had no missing/not applicable in any of the items related to the five factors).

^**b**^Standard deviation.

^**c**^ITOFC = Item-to-own-factor correlation.

^**d**^Reverse-scored items.

## Discussion

We have described the results of a validation study using the Norwegian translation of the SAQ-AV in the primary care setting, both in OOH clinics and regular GP practices. As far as we know, this is the first systematic study of patient safety culture in a representative sample of OOH clinics in Norway. In addition, the SAQ has not earlier been validated in a Norwegian GP setting.

Among those responding to the questionnaire, there was a low degree of missing values. The overall response rate was, however, not optimal. It was substantially higher, almost twice as high, among nurses compared to medical doctors. This difference is in accordance with findings from a SAQ-study in a Norwegian hospital setting [17]. A possible reason for this could be that nurses may feel closer linked to the clinical units than the doctors, since they spend a larger amount of their working time there. Many primary care doctors do clinical work different places (in GP practices, OOH clinics, nursing homes, well baby clinics), while most nurses are employed only in one clinical setting. This may imply an increased interest in creating a positive and safe working environment.

The response rate was higher among doctors in GP practices (55%) than doctors in OOH clinics (33%). As GPs commonly spend more working hours in GP practices than most OOH-doctors do in casualty clinics, the higher response rate increases the validity of the patient safety assessment in general practice. OOH-doctors commonly work in casualty clinics for only a limited part of their total working hours. This may lead to a poorer linkage to this particular work place, meaning that the rather low response rate should not reduce the validity of the patient safety assessment in OOH clinics very much. Nurses are more often employed only in one clinic, and spend most of their working time there. The high response rates among nurses both in GP practices (73%) and OOH clinics (71%), strengthen the validity of the patient safety assessments.

Nurses might have had the opportunity to fill in the questionnaire during their work time – whereas it was a free time, and thereby an unpaid, activity for the majority of the doctors. This may explain the higher interest among nurses for participating in a study of how their working environment supports patient safety. There also may be a gender difference in how to respond to the reminders that were sent. A majority of the responses tended to be skewed towards the favourable side of the scale, reflecting a positive attitude to patient safety.

The Cronbach alphas were above the recommended limit of 0.70 for four of the factors, and not much below for the fifth factor Perceptions of management (0.67). These values demonstrate internal consistency of the factors.

The five factor model (Teamwork climate, Safety climate, Job satisfaction, Working conditions and Perceptions of management) fitted the dataset from this Norwegian primary health care setting, with acceptable goodness-of-fit values. A model containing a sixth factor, Stress recognition – one of the original six SAQ factors – could not be confirmed. As mentioned above, several studies have found it to be invalid as an organizational climate scale, and that may explain our result.

## Conclusions

The results of our study indicate that the Norwegian translated version of the Safety Attitudes Questionnaire – Ambulatory Version, with the five confirmed factors, might be a useful tool for measuring several aspects of patient safety culture in the primary care setting. Discussing the results at unit level may facilitate strategies to reduce risk of medical errors, by focusing on improvement of quality and health care provider attitudes. In future studies, possible patient safety culture differences between OOH clinics and GP practices should be investigated. Likewise, it needs to be clarified whether different professional background may influence attitudes to patient safety in the primary care setting. Further research should also validate the questionnaire by correlating the scores on the SAQ-AV domains to patient-associated outcomes, not only medical errors and negative health outcomes, but also patient satisfaction and employee-related outcomes, such as staff satisfaction, retention and burnout.

## Competing interests

The authors declare that they have no competing interests.

## Authors’ contributions

GTB was responsible for designing the study, developing the Norwegian translated version of the SAQ-AV questionnaire, data collection, analysis and interpretation of data, and writing the manuscript. DH participated in developing the Norwegian SAQ-AV questionnaire, and was responsible for the statistical analysis and interpretation of results, in addition to revising the manuscript critically. EHH participated in designing the study, developing the Norwegian SAQ-AV questionnaire, data collection and revising the manuscript critically. ECTD participated in designing the study, developing the Norwegian SAQ-AV questionnaire, data analyses and interpretation, and writing the manuscript. All authors read and approved the final manuscript.

## Authors’ information

GTB, MD and PhD, is Professor in General Practice at the University of Bergen and Principal Researcher at the National Centre for Emergency Primary Health Care, Bergen, Norway. He is specialist in Family Medicine, and works clinically as General Practitioner in Bømlo, Norway.

DH is Professor at the Institute of Health and Society, Faculty of Medicine, University of Oslo. He is a social scientist.

EHH, MPH and PhD, is Associate Professor at Haraldsplass Deaconess University College of Bergen, Norway, and Research Adviser at The Norwegian Nurses Organization in Norway. She is a registered nurse.

ECTD, MD and PhD, is a Senior Researcher at the Health Services Research Unit at Akershus University Hospital and a Senior Advisor for the Norwegian Knowledge Centre, secretariat of the Norwegian patient safety campaign. She is a Consultant in internal medicine in an ambulatory clinic for rehabilitation of stroke patients, at Akershus University Hospital.

## Pre-publication history

The pre-publication history for this paper can be accessed here:

http://www.biomedcentral.com/1472-6963/14/139/prepub
